# Teacher-class relationship and emotional intelligence in the academic output and generic competence of higher education students

**DOI:** 10.1371/journal.pone.0292120

**Published:** 2023-10-13

**Authors:** Rahat Yasmeen, Ashfaque Ahmad Shah, Sehrish Naseer, Zunaira Fatima Syeda

**Affiliations:** 1 Department of Education, University of Sargodha, Sargodha, Pakistan; 2 Department of Educational Development, University of Baltistan, Skardu, Pakistan; Zhejiang University, CHINA

## Abstract

Academic output of students is considered as a crucial element and it is affected by various aspects. Teacher-class relationship, emotional intelligence and generic competence are the elements which can have a considerable effect on the academic output of the students. Current study was intended to find out the moderating role of teacher-class relationship, emotional intelligence in the academic output and generic competence of higher education students. All the teachers and students of University of Sargodha were the population of the study. The sample was taken from four departments of different faculties of University of Sargodha i.e., Faculty of Agriculture, Faculty of Medical and Health Sciences, Faculty of Arts and Humanities and Faculty of Social Sciences. For this study, a sample of 21 teachers and 320 students from regular and self-support programs was selected conveniently from University of Sargodha. For data collection, three adapted instruments were used. Findings of current study concluded an insignificant moderating role of teacher-class relationship and emotional intelligence in the academic output and generic competence of students at higher education. The results of present study provide guideline to curriculum developers for developing the generic competence of students so that they can progress in future prospects which will eventually make contribution towards economy, moreover, stakeholder may take steps in order to develop the emotional intelligence of students by providing them needful counseling and trainings which will improve the academic output of students.

## Introduction

Academic output of students has been effected by various aspects which has been discussed in literature; these aspects include teacher-class relationship, well-being, motivation, emotional intelligence and generic competence [1]. Teachers has a potential role in academic performance of students and teacher-class relationship has been considered as an important element [2]. Another important aspect in academic output of students is intelligence which is defined as the ability to think wisely. Emotional intelligence is defined as a capacity to control, express and handle the emotions accordingly as well as contributes significantly in health, academic life as well as career of the students [3].

The concept of "Emotional Intelligence" was first used by Salovey and Mayer in 1990. They defined it as "a type of societal intelligence which includes having the capacity to observe one’s own as well as other people’s emotions and feelings, as well as differentiate among them, and apply this information to direct one’s thinking and action [4].

Current theoretical model of emotional intelligence is known as model of abilities of Mayer and Salovery. It encompasses the four types of skills:

1. Accurately identify emotions
2. Access and create emotions to support thought
3. Comprehend emotions and emotional knowledge
4. Manage emotions introspectively in order to foster both emotional and intellectual development

Each ability in Mayer and Salovey’s paradigm is assessed using particular activities [5].

In higher education, the aim of equipping graduates with necessary skills is to promote the changes in curricula of university in order to work efficiently which will contributes towards development in society [1]. Teacher-Class Relationship can have positive or negative effect on Academic Output and life of students; behavior of teacher indirectly effects the academic output of students [6].

If a strained relationship has been observed between teacher and class, students are less motivated and less encouraged towards studies [7]. Literature indicated that various studies has been found observing the student emotions and teacher-class relationship [8]. Phenomena of teacher-class relationship and emotional intelligence has been investigated; teacher-class relationship has been studied in order to better understand the feelings of students [9]. Students who have good emotional intelligence can deal with challenges in a productive way [10]. Emotional intelligence has an impact on both mental and physical activity of students [11]. Emotional intelligence was discovered to be a major element in academic output by researchers [12].

Several significant research areas in the field of strategic management clearly demonstrate the core competence idea. These streams include resource-based [13, 14], competence-based [15], learning-based [16], knowledge-based [17], & adaptive capability-based views [18, 19]. According to [20], the premise of generating competency through higher education has been defended. The traditional viewpoint has been changed because of the professional courses offered by the universities; more courses are being introduced in university to improve generic competence [21]. Universities cannot guarantee that their students will be equipped with all of the necessary generic competencies stated forth in curriculum, as [22] pointed out. Various studies has been conducted in order to improve the student performance and solve their concerns [23]. Few studies have been conducted on the teacher-class interaction and the role of emotional intelligence in academic output [24]. Association between academic output and emotional intelligence of students was observed by [25]. Teachers and policymakers are increasingly acknowledging that emotional intelligence is critical in developing skills of students for their future lives and careers [26]. Universities are responsible for assessing the learning outcomes including competency growth [20]. Nowadays, university education is focused on the development and acquisition of generic abilities which are known as the general traits and abilities which are required for personal and professional development as well as the removal of barriers towards acquisition of generic skills [27]. Teachers need generic skills because they allow students to become more analytical and self-directed [28].

In the United States, grading academic performance has been a practice since the 1830s. In Boston, Massachusetts, educational reformers Horace Mann and Samuel Gridley Howe evaluated student progress using a standardized test. With the Kansas Silent Reading Test, developed by Kansas school administrator Frederick J. Kelly in 1914, the concept of standardized testing was advanced [29]. Academic output is defined as the academic achievement of students which is measured in terms of grades had an impact on educational policies in terms of preventing educational difficulties [30]. Present study will contribute in various ways. First, this study will add to the literature in context of role of Teacher-Class Relationship and Emotional Intelligence in the Academic Output and Generic Competence of Higher Education Students which is beneficial for the development of student’s performance. Secondly, students will gain a comprehensive understanding of their academic life challenges, through identification they will find ways to resolve them. Thirdly, this study will benefit the teachers in higher education since it will make them aware of the importance of the teacher-class relationship and by using adaptable behavior, they may improve the performance of students. Finally, students will have awareness of their own emotional intelligence and competence issues, they will be better able to manage the stress and improve their academic performance consequently.

Purpose of the current study is to explore the role of teacher-class relationship and emotional intelligence in academic output and generic competences of students in higher education. Academic achievement of students is a primary objective of education and it is required to compete in professional life. There are various factors which can affect the academic performance. Evidences from the literature demonstrated that teacher-class relationship can affect the performance of students. Similarly, it is evident that emotional intelligence can play its role in improving generic competence; literature offered mixed results. To best of our knowledge, there is no study found in literature which investigated the current phenomena. Therefore, current study intended to identify the role of teacher-class relationship and emotional intelligence in academic output and generic competences of students in higher education.

This study contributes to understanding the role of teacher-class relationship and emotional intelligence in academic output and generic competences of students in higher education. We first identify the role of role of teacher-class relationship and emotional intelligence in academic output. Furthermore, we will investigate the role of teacher-class relationship and emotional intelligence in generic competences of students in higher education.

In literature, various studies are found examining the factors which can affect the academic output of the students in higher education. Present study is significant contribution in existing literature on factors which can affect the performance of the students. Secondly, to best of our knowledge, there is no study found which investigated these factors for improving the academic output and generic competence of students. Thirdly, current study will contribute to the policy recommendation for suggesting ways in order to improve academic achievement as well as generic competence of the students at higher education.

Objectives of the current study are as follows:

1. To find out the relationship of students’ teacher-class relationship and emotional intelligence on academic output and generic competence
2. To find out the effect of students’ teacher-class relationship and emotional intelligence on academic output and generic competence
3. To find out the moderating role of students’ emotional intelligence in the relationship between teacher- class relationship, students’ academic output and students ‘generic competence
4. To find out the moderating role of teacher-class relationship in the relationship between students’ emotional intelligence, generic competence and academic output

## Methodology

Quantitative research method was used in the research; study was descriptive in nature. Cross-sectional survey research design was used for current study.

### Population of the study

All the students and teachers of University of Sargodha was included in the population of the current study.

### Sampling and sample

For the present study, convenience sampling method was used. Sample of the current study includes the students and teachers from the faculty of Arts and Humanities, Faculty of Social Sciences, University College of Agriculture and Sargodha Medical College. Four departments from these faculties have been selected conveniently; these departments include Department of English, Department of Social Work, Department of Plant Pathology and Sargodha medical college. By using convenience sampling technique, sample of current study included 21 teachers and 320 students; 30 students were selected from regular and self-support BS programs (5th and 7th semester) of each selected department and students of 3rd and 4th year of Sargodha Medical College conveniently while 5 teachers from each department were selected; we have received one extra response from Sargodha Medical College which was included in this study.

### Research instruments

Three Adapted questionnaires were used for the present study.

Wong and Law Emotional Intelligence Scale (WLEIS; [31]: The scale consists of 16 items, and the answers were obtained according to a seven-point Likert scale, ranging from 1 (strongly disagree) to 7 (strongly agree) to measure the student’s perception of their EI, distributed among four dimensions: self-emotional appraisal (SEA), 4 items (α = .774; e.g., I have a good sense of why I feel certain feelings most of the time); other emotional appraisal (OEA), 4 items (α = .608; e.g., I always know my friends’ emotions from their behavior); use of emotion (UOE), 4 items (α = .646; e.g., I am a self-motivating person; and regulation of emotion (ROE), 4 items (α = .813; e.g., I have good control of my emotions). The internal consistency for the all scale in this study is α = .836. Teacher-Class Relationship Inventory (TCRI; [32]): The scale consists 14 statements, and the responses were taken according to a seven-point Likert scale, ranging from 1 (strongly disagree) to 7 (strongly agree) to measure the teacher’s perception of their teacher-class relationship. The internal consistency for the all scale in this study is α = .737. Generic Competences Scale (GCS; [33]): The scale consists 19 statements, and the responses were taken according to a seven-point Likert scale, ranging from 1 (strongly disagree) to 7 (strongly agree) to measure the student’s perception of their generic competences. The internal consistency for the all scale in this study is α = .690. Academic output was measured through marks (Annual system) and CGPA (semester system) of students.

### Data collection

Current study was the requirement of masters of philosophy in education, therefore as per the criteria of institution, present study was approved by the board of studies. Moreover, there was no requirement of approval from the ethical committee. Researchers approached the participants by targeting the relevant discipline and department as per the pre-defined criteria. Included students were selected as per the requirement of sample of the current study. Participants consent was taken before the study by ensuring that their information will be kept confidential and will be not use for any other purpose.

Data was collected online through Google form because of the pandemics COVID-19, educational institutions were on online mode.

### Validity

For the purpose of validation, instruments were discussed with six teachers (for expert opinion) of department of education, University of Sargodha, Sargodha. After expert opinion, unclear and irrelevant statements were removed from the instruments. After validation of instruments, pilot testing was conducted by using data of 24 students and 2 teachers.

### Data analysis

Missing data has been handled by the researchers by setting privacy setting to google form as data was collected through google form, researcher fixed the privacy setting that participants were required to fill every item; by skipping an item, there was no chance to jump on the next one.

Descriptive statistics and inferential statistical test were used for data analysis. For the analysis of data, normality test was applied to determine the distribution of data. Pearson Product-Moment correlation analysis was used in order to find out the relationship among Teacher-Class Relationship, Emotional Intelligence, Academic Output and Generic Competence. Researcher applied linear regression analysis and multiple regression analysis to find out the effects of Teacher-Class Relationship and students’ Emotional Intelligence on academic output and Generic Competence of the students. Moderation analysis technique was used to find out the moderating role of Teacher-Class Relationship and Emotional Intelligence in the Academic Output and Generic Competence of students in higher education.

### Ethics statement

According to the local legislation and institutional requirements, ethical review and approval was not required for the study on human participants. But while preparing this manuscript, all other ethical considerations were fulfilled and consent of participants were taken in written form; therefore respondents (only volunteers) were informed and engaged for data collection through the written consent. Researchers ensured the respondents that this research is not harmful for anyone and will be used only for research purpose and their information will be kept confidential. Respondents were also had freedom to leave research at any stage if they feel unsatisfied.

## Results

### Descriptive statistics

Table 1 showed the frequency distribution of respondents with respect to gender, department and semester. It is indicated that 58.3% were female while 36.9% were male respondents. Moreover, it is showed that 22.35% respondents were from department of plant pathology, 30.7% respondents were from department of English, 21.4% were from Sargodha Medical College and 20.8% respondents were from department of Social Work.

**Table 1:** Gender, department and semester wise frequency distribution of respondents.

Gender	Frequency	Percentage	Department	Frequency	Percentage	Semester	Frequency	Percentage
Female	196	58.3	Plant Pathology	75	22.3	5th	155	46.1
Male	124	36.9	English	103	30.7	7th	165	49.1
			Medical College	72	21.4			
			Social Work	70	20.8			

### Test of normality

The main focus of the normality test is on skewness and kurtosis in latent trait distribution. Skewness and kurtosis can affect latent trait distribution. In the descriptive analysis of normality tests, conditions used for skewness and kurtosis as; normal → skewness = 0, kurtosis = 0 (means no skewness and no kurtosis), near normal → skewness = 0.25 kurtosis = 0.75, (means slight skewness and slight kurtosis), moderately nonnormal → skewness = 0.75 kurtosis = 1.75 (means moderate skewness and moderate kurtosis), highly nonnormal → skewness = 1.25 kurtosis = 2.75(means high skewness and high kurtosis) and extremely nonnormal → skewness = 1.50 kurtosis = 3.75(means extreme skewness and extreme kurtosis).

Normality analysis of teacher-class relationship showed that teacher class-relationship is near no normal; as skewness = .76 means that it is moderately skewed and kurtosis = -1.42 means moderately kurtosis. Results of normality analysis of student’s Emotional Intelligence indicated that emotional intelligence is moderately near to normal as skewness = -.77means that it is moderately negatively skewed and kurtosis = .53 means near slight negatively kurtosis. Deductions of normality analysis of students’ Academic Output showed that academic output is normal; as skewness = -.03 means that it is slight negatively skewed and kurtosis = -.07 means slight negatively kurtosis. Normality analysis of Generic Competence of students showed that generic competence is moderately normal as skewness = -.81 means that it is high moderately negatively skewed and kurtosis = .58 means more normally negatively kurtosis.

### Correlation analysis

Table 2 showed the relationship among *Teacher-Class Relationship*, *Emotional Intelligence*, *Academic Output and Generic Competence* of the respondents. Pearson correlation coefficient indicated a negative weak relationship between teacher-class relationship and emotional intelligence of respondents; which was statistically insignificant (*r* = -.03 *P* = .58). It means no relationship was found among teacher-class relationship and emotional intelligence of respondents. In relationship between Teacher-Class Relationship and Emotional Intelligence of student’s, we failed to reject the null hypothesis "there is no significant relationship between Teacher-Class Relationship and students Emotional Intelligence".

**Table 2:** Relationship among teacher-class relationship, emotional intelligence, generic competence and academic output of students.

		EI	AO	GCS
**TCR**	Pearson Correlation	-.030	-.184**	-.062
	Sig. (2-tailed)	.588	.001	.271
	N	320	320	320
**EI**	Pearson Correlation		-.012	.804**
	Sig. (2-tailed)		.836	.000
	N		320	320
**AO**	Pearson Correlation			.015
	Sig. (2-tailed)			.790
	N			320

** Correlation is significant at the 0.01 level (2-tailed)

Pearson correlation coefficient showed negative weak relationship among teacher-class relationship and academic output of respondents; which was statistically significant (r = -.18, *P* = .00). It means weak negative relationship exists among teacher-class relationship and academic output of respondents. In relationship between Teacher-Class Relationship and Academic Output of student’s, we rejected the null hypothesis "there is no significant relationship between Teacher-Class Relationship and Academic Output of students".

Results showed negative relationship between teacher-class relationship and generic competence of respondents which was statistically insignificant (r = -.06 *P* = .27). It means that there is no relationship between teacher-class relationship and generic competence of respondents. In relationship between Teacher-Class Relationship and Generic Competence of student’s, we failed to reject the null hypothesis "there is no significant relationship between Teacher-Class Relationship and Generic Competence of students".

Results presented the negative weak relationship between emotional intelligence and academic output of respondents which is statistically insignificant (*r* = -.01 *P* = .83). It showed that no relationship exists between student’s emotional intelligence and their academic output. In relationship between student’s Emotional Intelligence and their Academic Output, we failed to reject the null hypothesis "there is no significant relationship between student’s Emotional Intelligence and their Academic Output".

Deductions revealed a strong positive relationship between emotional intelligence and generic competence of respondents which is statistically significant (*r* = .80 *P* = .00). It means that there is strong positive relationship between student’s emotional intelligence and their generic competence. In relationship between student’s Emotional Intelligence and their Generic Competence, we rejected the null hypothesis "there is no significant relationship between student’s Emotional Intelligence and their Generic Competence".

Results indicated a weak positive relationship between academic output and generic competence of students which is statistically insignificant (*r* = .01 *P* = 79). It presented that there is no relationship between student’s academic output and their generic competence. In relationship between student’s Academic Output and their Generic Competence, we rejected the null hypothesis "there is no significant relationship between student’s Academic Output and their Generic Competence".

### Regression analysis

Table 3 showed that coefficient regression, which implies that parameter of model, is significant. For the coefficients Table 2, researcher observed that teacher-class relationship parameter that is, β is significant with P value is .001. Given the coefficients (β = -.184 ≠ 0) with beta not equal to zero (0 coefficients mean that value of dependent variable does not consistently differ as the value of independent variable increases), therefore researcher rejected the null hypothesis "there is no statistically significant effect of Teacher-Class Relationship on Academic Output of Students"

**Table 3:** Effect of teacher-class relationship, emotional intelligence, generic competence on academic output, teacher-class relationship on generic competence of student, and students emotional intelligence on generic competence.

Coefficients					
Model	Unstandardized Coefficients		Standardize Coefficients	*T*	Sig.
	Beta	St. Error	*Β*		
(Constant)	112.458	11.967		9.397	.000
TCR	-.569	.170	-.184	-3.341	.001
(Constant)	72.745	1.295		56.182	.000
EI	-.003	.016	-.012	-.208	.836
(Constant)	72.184	1.170		61.675	.000
GCS	.003	0.12	.015	.267	.0
(Constant)	153.031	55.589		2.753	.006
TCR	-.872	.791	-.062	-1.103	.271
(Constant)	9.573	3.526		2.715	.007
EI	1.030	.043	.804	24.076	.000

The Table 3 showed that coefficient regression which implies that parameter of model is insignificant. For the above coefficients table, researcher see that emotional intelligence parameter, that is, *b* is insignificant with *P* value is .836. Given the coefficients (*β* = -.012 ≠ 0) with beta not equal to zero (0 coefficients mean that value of dependent variable does not consistently differ as the value of independent variable increases), therefore researcher failed to reject the null hypothesis” there is no statistically significant effect of student’s emotional intelligence on their Academic Output".

The Table 3 showed that coefficient regression which implies that parameter of model is insignificant. For the above coefficients table, researcher see that generic competence parameter that is, *β* is insignificant with *P* value is .790. Given the coefficients (*β* = .015 ≠ 0) with beta not equal to zero (0 coefficients mean that value of dependent variable does not consistently differ as the value of independent variable increases), therefore researcher failed to reject the null hypothesis" there is no statistically significant effect of students Generic Competence on their Academic Output".

Table 3 showed that coefficient regression which implies that parameter of model is insignificant. For the above coefficients table, researcher see that teacher-class relationship parameter that is, *β* is insignificant with *P* value is .271. Given the coefficients (*β* = -.062 ≠ 0) with beta not equal to zero (0 coefficients mean that value of dependent variable does not consistently differ as the value of independent variable increases), therefore researcher failed to reject the null hypothesis "there is no statistically significant effect of Teacher-Class Relationship on Generic Competence of students".

Table 3 indicated that coefficient regression which implies that parameter of model is significant. For the above coefficients table, researcher see that emotional intelligence parameter, that is, *β* is insignificant with *P* value is .000. Given the coefficients (*β* = .804 ≠ 0) with beta not equal to zero (0 coefficients mean that value of dependent variable does not consistently differ as the value of independent variable increases), therefore researcher rejected the null hypothesis " there is no statistically significant effect of student’s Emotional Intelligence on their Generic Competence".

The Table 4 showed that coefficient regression which implies that parameter of model is insignificant. For the above coefficients table, researcher see that teacher-class relationship, parameter, that is, *β* is significant with *P* value is .001, the emotional intelligence parameter, that is, *β* is insignificant with *P* value is .541 and the generic competence parameter, that is, *β* is insignificant with *P* value is .596. Given the coefficients (*β* = -.183, *β* = -.057, *β* = -.049 ≠ 0) with beta not equal to zero (0 coefficients mean that value of dependent variable does not consistently differ as the value of independent variable increases), therefore researcher failed to reject the null hypothesis "there is no statistically significant effect of Teacher-Class Relationship, Emotional Intelligence and Generic Competence on Academic Output of students".

**Table 4:** Effects of teacher-class relationship, emotional intelligence and generic competence on academic output of students.

Coefficients					
Model	Unstandardized Coefficients		Standardize Coefficients	*t*	Sig.
	Beta	St. Error	*Β*		
(Constant)	112.452	12.141		9.262	.000
TCR	-.565	.171	-.183	-3.301	.001
EI	-.016	.026	-.057	-.613	.541
GCS	.011	.020	-.049	.531	.596

The Table 5 showed that coefficient regression which implies that parameter of model is significant. For the above coefficients table, researcher see that teacher-class relationship parameter that is, *β* is insignificant with *P* value is .264, the emotional intelligence parameter that is, *β* is significant since the *P* value is .000. Given the coefficients (*β* = -.037, *β* = .802 ≠ 0) with *β* not equal to zero (0 coefficients mean that value of dependent variable do not consistently differ as the value of independent variable increases), therefore researcher rejected the null hypothesis" there is no statistically significant effect of Teacher-Class Relationship and Emotional Intelligence on Generic Competence of students".

**Table 5:** Effects of teacher-class relationship and emotional intelligence on student’s generic competence.

Coefficients					
Model	Unstandardized Coefficients		Standardize Coefficients	*t*	Sig.
	Beta	St. Error	*β*		
(Constant)	46.756	33.430		1.399	.163
TCR	-.527	.471	-.037	-1.118	.264
EI	1.029	.043	.802	24.041	.000

### Moderation analysis

Table 6 revealed that teacher-class relationship was insignificantly related to student’s academic output; and emotional intelligence insignificantly moderated the relationship between teacher-class relationship and student’s academic output as the interaction effect i.e., that teacher-class relationship ×emotional intelligence → academic output (*β* = -.003, *P* = .674) was insignificant. We failed to reject the null hypothesis " there is no statistically significant moderating role of students Emotional Intelligence in the relationship between Teacher-Class Relationship and students’ Academic Output".

**Table 6:** Moderating role of students emotional intelligence in the relationship between teacher-class relationship and students’ academic output, students emotional intelligence in the relationship between teacher-class relationship and students generic competence, teacher-class relationship in the relationship between students emotional intelligence and academic output, and teacher-class relationship in the relationship between students emotional intelligence and generic competence.

	β	*p*	*LLCI*	*ULCI*	*R* ^*2*^	*R*^*2*^ *Change*
Constant	94.2987	.0411	3.8527	184.7448		
TCR	-.3061	.6384	-1.5866	.9744	.0348	.0005
EI	.2301	.6807	-.8690	1.3292		
TCR×EI	-.0033	.6742	-.0189	.0122		
Constant	-158.7885	.2106	-407.8287	90.2516		
TCR	2.3834	.1845	-1.1423	5.9091	.6503	.0031
EI	3.6170	.0193	.5907	6.6433		
TCR× EI	-.0367	.0933	-.0795	.0062		
Constant	94.2987	.0411	3.8527	184.7448		
EI	.2301	.6807	-.8690	1.3292	.0348	.0005
TCR	-.3061	.6384	-1.5866	.9744		
EI× TCR	-.0033	.6742	-.0189	.0122		
Constant	-158.7885	.2106	-407.8287	90.2516		
EI	3.6170	.0193	.5907	6.6433	.6503	.0031
TCR	2.3834	.1845	-1.1423	5.9091		
EI× TCR	-.0367	.0933	-.0795	.0062		

Results of Table 6 represented that teacher-class relationship was insignificantly related to their generic competence; and emotional intelligence insignificantly moderated the relationship between emotional intelligence and student’s generic competence as the interaction effect i.e., that teacher-class relationship ×emotional intelligence → generic competence (*β* = -.036, *P* = .093) was insignificant. We failed to reject the null hypothesis "there is no statistically significant moderating role of students Emotional Intelligence in the relationship between Teacher-Class Relationship and students Generic Competence".

Table 6 described that emotional intelligence was insignificantly related to their student’s academic output; and teacher-class relationship insignificantly moderated the relationship between teacher-class relationship and student’s emotional intelligence as the interaction effect i.e., that emotional intelligence × teacher-class relationship → academic output (*β* = -.003, *P* = .674) was insignificant. We failed to reject the null hypothesis " there is no statistically significant moderating role of Teacher-Class Relationship in the relationship between students Emotional Intelligence and Academic Output.".

According to the results of Table 6, emotional intelligence was insignificantly related to their generic competence; and teacher-class relationship insignificantly moderated the relationship between teacher-class relationship and student’s emotional intelligence as the interaction effect i.e., that emotional intelligence × teacher-class relationship → generic competence (*β* = -.036, *P* = .093) was insignificant. We failed to reject the null hypothesis; "there is no statistically significant moderating role of Teacher-Class Relationship in the relationship between students’ Emotional Intelligence and their Generic Competence".

## Discussion

This study intended to observe the “Role of Teacher-Class Relationship and Emotional Intelligence in the Academic Output and Generic Competence of Students in Higher Education”. Deductions of the research described that teacher-class relationship had a significant effect on academic output of students.

Supporting the results of current study, literature showed that teachers have a significant role in academic life of students. Teacher-class relationship is different than student’s relationship with their family, friends and fellows; teacher-class relationship has much effect on student’s performance than student-student relationship [34]. Correspondingly, in support of current study, results showed that teacher-class relationship is considered as a crucial aspect in performance of students [35]. Results of prior study are in line with findings of current study which claimed that teacher-class relationship influence the ideas, expectations toward academic achievement and wellbeing of students [36].

Supporting the argument of present study, it is showed that smooth teacher-class relationship motivates the students towards participation in discussion and classroom activities which enable them to achieve good grades; in this way teacher-class relationship effect the academic performance of students [37]. Teachers plays an important role in student’s life and teacher’s behavior have much influence on life of students; teacher’s relationship with their students can make or break the mental health and personality of students [38].

Result of prior study are contradictory to the findings of this study which revealed that there was an insignificant moderating role of teacher-class relationship in relationship between students’ academic output and generic competence. Conversely, results of a study showed that teacher-student association has a significant indirect moderating effect on mathematical self-efficacy, but insignificant direct effect on mathematical ability [39]. Results of current study described that emotional intelligence have an insignificant effect on student’s achievement; insignificant moderating role of student’s emotional intelligence in the relationship between students’ academic output and generic competence was observed. Results of prior study are in line with findings of current study which indicated that emotional intelligence had an insignificant effect in relation among parenting approach and academic output [40]. Opposing the results of current study, [41], discussed that emotional intelligence had a significant moderating role in relation between deviant behavior and academic achievement. Supporting the results of current study, prior study showed the insignificant association among emotional intelligence and academic output but results of other studies are contradictory as they supported the significant connection among emotional intelligence and academic output [42]. Result of past study opposed the findings of current study, positive significant relationship is found between emotional intelligence and academic output.; academic output is considered as function of emotional intelligence [43]. Opposing the results of current study, it is described that emotional intelligence is helpful for students in achieving their academic goals [44]. Correspondingly, result of prior study opposed the findings of current study showed that emotional intelligence plays a great role in the academic output; high emotional intelligence of the students helps them to manage their life and motivate for better achievements in their academic career [45]. Findings of the study showed that generic competence have an insignificant effect on academic output of students. While studies supported that generic competence is necessary for better understanding and achievement of educational goals [46]. Now a days, education pays much attention on acquisition of generic competencies in students rather than on traditional educational styles for betterment of students and achievement of academic grades [47].

Result of present study are in line with findings of prior study which indicated that effective teacher-student communication, teachers’ creation of an emotionally open learning environment, teachers’ demonstration of true equity, and teachers’ demonstration of mutual respect have a significant impact on students’ academic performance in mathematics at Junior Secondary School III in Nigeria [48]. Supporting the results of current study, empirical research has demonstrated that antagonistic instructor actions, such as embarrassing pupils or discounting their contributions, demotivate students and cause them to feel discouraged, dissatisfied, or helpless [49]. Results of present study are in line with a prior study which indicated that these results confirmed that found a moderate relationship between EI levels and academic success [50]. A moderate relationship has been found between Emotional Intelligence and academic performance of students [51].

According to the result of prior study, higher degrees of dedication and compassion were found to be positively correlated with emotional intelligence, and this, in turn, was found to improve academic achievement [52].

## Conclusions

The main purpose of current study was to observe the “Role of Teacher-Class Relationship and Emotional Intelligence in the Academic Output and Generic Competence of Higher Education Students”. Results of present study revealed that data was normally distributed. Moreover, the results of current study presented an insignificant relationship between teacher-class relationship and emotional intelligence, teacher-class relationship and academic output, teacher-class relationship and generic competence, student’s emotional intelligence and their academic output and student’s academic output and their generic competence. Deductions of present study revealed a significant relationship between student’s emotional intelligence and their generic competence. This study will provide ways to improve generic competence by improving emotional intelligence which can be beneficial for students in many ways. Results presented that there was a significant effect of teacher-class relationship on academic output of students, students emotional intelligence on generic competence of students. Students will improve their academic performance by making better their relationships with their teachers; teachers can also play their role in strengthen the relationship with students which can improve their performance consequently. Results presented an insignificant effect of student’s emotional intelligence on academic output, student’s generic competence on academic output and teacher-class relationship on generic competence of students. Results of current study described an insignificant effect of teacher-class relationship, emotional intelligence and generic competence on academic output of students. Results of study revealed a significant effect of teacher-class relationship and emotional intelligence on generic competence of students. Students can improve their competences if they will be able to improve their emotional intelligence and teacher-class relationship; by improving their generic competence, students can perform better. Results of the present study showed that there was an insignificant moderating role of student’s emotional intelligence in the relationship between teacher-class relationship and students’ academic output, student’s emotional intelligence in the relationship between teacher-class relationship and student’s generic competence, teacher-class relationship in the relationship between student’s emotional intelligence and academic output and teacher-class relationship in the relationship between student’s emotional intelligence and generic competence.

### Limitations

1. The present study included only four faculties from University of Sargodha, Sargodha. The future study could be expanded by including other faculties of University of Sargodha, Sargodha.
2. Study major intends was to find “Role of Teacher-Class Relationship and Emotional Intelligence in the Academic Output and Generic Competence of Higher Education Students”. But in further studies we can see the mediating effect of emotional intelligence in relation with satisfaction or some other variables on academic achievement.

### Implications

- The results of present study provide guideline to curriculum developers for developing the generic competence of students so that they can progress in future prospects which will eventually make contribution towards economy.
- Deductions of current study offer favorable results for teachers by using stakeholders can take steps in order to improve the teacher-class relationship so that students can perform better.
- Stakeholder may take steps in order to develop the emotional intelligence of students by providing them needful counseling and trainings which will improve the academic output of students.
